# Editorial: The Campbell Crime & Justice Coordinating Group: Celebrating 20 years of achievements

**DOI:** 10.1002/cl2.1099

**Published:** 2020-06-29

**Authors:** Lorraine Mazerolle, Peter Neyroud

The Crime and Justice Coordinating Group (CJCG) was one of the founding coordinating groups of the Campbell Collaboration, created at a meeting of more than 80 persons from 12 countries in Philadelphia in February 2000. Farrington and Petrosino (2001) describe the broad mission of the CJCG as overseeing the preparation, maintenance, and accessibility of systematic reviews of research on the effects of criminological and criminal justice interventions, with the primary emphasis on reviews of interventions designed to prevent or reduce crime or delinquency. In this Editorial we offer some insight into the breadth and depth of the CJCG activities over the last 20 years, highlighting some of the most highly cited reviews and their policy influences across the globe.

Since inception, the CJCG has shepherded 52 full reviews through the full Campbell process. The population of systematic reviews examines a broad array of research on the effects of interventions delivered by the courts, police, probation or parole agencies, prisons, and community groups. In particular, the CJCG has striven to build funded programs of research to create clusters of systematic reviews, receiving funds from ten different funding sources from the US (Arnold Ventures, National Institute of Justice, Department of Homeland Security, Jerry Lee Foundation), UK (National Policing Improvement Agency, Home Office), Canada (Public Safety Canada, Department of Justice Statistics), Switzerland (Jacob's Foundation), and the Swedish National Council for Crime Prevention. The CJCG has raised approximately USD$3.5 million over the last 20 years to fund these cluster programs of reviews, principally to cover the direct costs of conducting extensive searches of the published and gray literatures, screening hundreds and thousands of titles and abstracts to identify high‐quality studies to be included in our reviews. This type of funding, particularly for multi‐year clusters of reviews, provides long term stability and the capacity of the CJCG to build critical mass of highly skilled review teams from around the world.

Over the last 20 years, the CJCG has published 52 full, final report systematic reviews. The CJCG has worked hard to maintain a healthy pipeline of reviews, soliciting, and supporting a range of different review teams to submit titles and protocols. We now have an average of five new titles being approved by the CJCG every year, 4 protocols being approved and published every year and at least 3 full reviews being completed each year, with some reviews taking between 2 and 3 years to complete. Generating this pipeline of work has been a focus for the CJCG since the early establishment years. Indeed, until 2006, just two reviews had been completed with the early work of the group concentrated on building up expertise in teams to submit titles and protocols and then working closely with the review teams to get the reviews to final report stage.

The number of reviews being published in any one year is highly correlated in line with the cluster of funding being provided to the group. For example, in 2008 alone nine reviews were published, in 2012 we published seven reviews and in 2019 we published six reviews. The National Policing Improvement Agency cluster program of reviews, for example, funded 10 reviews in the area of policing. The recent funding from the US Department of Homeland Security and Public Safety Canada to fund at least 25 reviews over a 5‐year cluster of funding around terrorism and national security creates unprecedented opportunities to advance evidence‐based policy and practice in the area of national security. Our general point is that direct, multi‐year clusters of funding for cohesive programs of systematic reviews are a major factor in our capacity to advance knowledge about the evidence pertaining to crime and justice interventions.

By far the largest number of reviews published by the CJCG has been in the area of policing (*N* = 23, 44% of all reviews) on interventions such as hotspots policing, problem‐oriented policing, pulling levers, legitimacy policing. We have also published 11 crime prevention program reviews (21%, including neighborhood watch, CCTV), 12 criminal justice treatment program reviews (including reviews in the areas of cognitive‐behavioral therapy, sex offender treatments), 3 correctional reviews (including boot camps) and 3 courts reviews (including drug courts).

The CJCG aims to build a program of routine updates of completed reviews. So far, we have published seven updated reviews on topics such as hot spots policing, pulling levers policing, and incarceration based drug treatment on criminal behavior. One additional update review on problem‐oriented policing is published in this issue (Hinkle, Weisburd, Telep, & Petersen, 2020). These updates are incredibly important: with the exponential increase in high‐quality primary evaluation studies in the crime and justice arena (see, for example, Mazerolle, Eggins, Higginson, & Stanko, 2017), there is a policy imperative for facilitating review teams to update their reviews is a timely manner.

Finally, at its core, Campbell and the CJCG aims to use evidence from our suite of products to influence policy and practice. The initiative of the Campbell Collaboration to produce plain language summaries (PLS) of completed reviews is fully embraced by the CJCG. These PLS are written for policy and practice audiences, they are well produced with striking color photographs and graphics and include clearly explained, basic take‐home messages from the reviews. To date, 23 of the 52 reviews published in the CJCG library have been translated into PLS. Our tracking of the policy influences of the CJCG reviews and PLS shows that our products have been used to encourage local testing of interventions that have been shown to work (e.g. Hotspots Policing review being used in Trinidad and Tobago and across Latin America); used by UK National Police Chief's Council and the Director of Public Prosecutions to expand the use of police‐initiated diversion for young people (see Wilson, Brennan, & Olaghere, 2018); formed the basis of policy advice to the Dutch Ministry of Justice on the use of custodial and non‐custodial sentencing (see Villettaz, Killias, & Zoder, 2006); referenced in the US President's Task Force on 21^st^ Century Policing as evidence to support pillar one: building trust and legitimacy (Mazerolle, Bennett, Davis, Sargeant, & Manning, 2013); and, more generally, our collection of reviews have informed policy discussion by the Scottish Government, the Ministry of Security in the Netherlands, the Swedish Ministry of Justice, the Indian Ministry of Home Affairs and the Council of Canadian academies.

As we write this Editorial in April 2020, at the height of the COVID‐19 pandemic across the globe, we are working hard with justice agencies across the world to help criminal justice agencies use sound, scientific evidence to deal with unprecedented pressures on their frontline resources. Being a key part of the International Campbell Collaboration means that we are also able to draw on research across the wider disciplines within the Campbell family to enable us to help with our mission of providing better evidence for a better world. Stay well.
